# Determinants of folic acid intake during preconception and in early pregnancy by mothers in Ibadan, Nigeria

**DOI:** 10.11604/pamj.2014.19.113.4448

**Published:** 2014-10-01

**Authors:** Taiwo Akeem Lawal, Amos Olufemi Adeleye

**Affiliations:** 1 Division of Paediatric Surgery, Department of Surgery, College of Medicine, University of Ibadan, and University College Hospital, Ibadan, Nigeria; 2 Division of Neurological Surgery, Department of Surgery, College of Medicine, University of Ibadan, and Department of Neurological Surgery, University College Hospital, Ibadan, Nigeria

**Keywords:** Periconceptional, folic acid, neural tube defects, prevention, determinants

## Abstract

**Introduction:**

In order to identify targets for primary preventive strategies, we explored possible predictors of periconceptional folic acid (pFA) intake in a Nigerian population of reproductively active women.

**Methods:**

A cross sectional study of mothers attending immunization clinics at two hospitals was conducted between May and November 2012. Information obtained included sociodemographic and obstetric details and periconceptional usage of FA. Independent variables were analysed as predictors of pFA intake using chi-square statistical test and multinomial logistic regression.

**Results:**

The study involved 602 mothers aged 17 to 42 years; 23% had a university degree and 66% were in the working class. Preconceptional usage was proven in only 15 (2.5%). Periconceptional usage was more likely among professionals (X^2^ = 41.194, p < 0.001), have university degree (X^2^ = 53.089, p < 0.001), be primigravid (X^2^ = 18.415, p < 0.001) and early antenatal clinic attendees (X^2^ = 355.9, p < 0.001). Women were less likely to know that FA could prevent birth defects if in the working class (1.7% vs. 11.1%, X^2^ = 25.593, p < 0.001), less educated (0.5 vs. 10.9%, X^2^ = 38.083, p < 0.001) or booked late for antenatal care (2.0 vs. 5.9%, X^2^ = 5.767, p = 0.016). The determinants of late commencement of FA were low social class (OR = 4.29, 95% CI: 1.59, 11.31), lack of university education (OR = 4.58, 95% CI: 3.06, 6.87) and late booking (OR = 104.27, 95% CI: 53.09, 204.76).

**Conclusion:**

In this population of reproductively active women, pFA intake and knowledge of its health benefits are poor amongst mothers - in working class, with limited education, and who present late for antenatal care.

## Introduction

The incidence of neural tube defects (NTDs) in developing countries has been reported to be up to four-fold higher than in developed ones [1, 2]. The life-long medical and socio-economic consequences of NTDs in affected children are equally known to be worse in low-resource settings. Folic acid (FA) taken by women at least four weeks prior to conception and throughout the first trimester of pregnancy, or periconceptional FA intake, has been associated with up to 80% reduction in the incidence of NTDS [3, 4]. Early exposure in pregnancy to FA has also been found beneficial in the prevention of some other reproductive mishaps like congenital heart diseases, cleft lip and palate, limb defects and anorectal malformations [5, 6]. In developing countries, the absence of pre-conceptional counselling coupled with sub-optimal antenatal care services is a barrier to reducing the incidence of NTDs [7]. In order to identify targets for primary preventive strategies, we aimed to ascertain factors associated with periconceptional intake of FA as well as the level of the knowledge of its role in the prevention of birth defects in a selected Nigerian population of reproductively active women. It was also hoped that this would give insights into the possible predictors of late commencement of FA by this study population.

## Methods

A descriptive cross sectional study of mothers attending postnatal immunization clinics at two hospitals in Ibadan, Nigeria, was conducted between May and November 2012. The first health facility is a general hospital administered by the state government, which caters for citizens majorly from the low socioeconomic groups in the state capital. The second one is a tertiary hospital operated by the federal government with its clientele from all strata of the society. The two hospitals, with the highest delivery rates in the state, were selected to have a sample containing mothers from the different strata of the population. Following ethical approval from the Joint University of Ibadan/University College Hospital Ethical Review Committee, structured, pretested, interviewer-administered questionnaires were used to obtain information from 602 consenting mothers consecutively selected at the two hospitals using simple random sampling technique. A table of random numbers was used and a list of mothers who signed the attendance list in the morning of the clinic used to generate the sampling frame. A minimum sample size of 220 women from each hospital, i.e. total of 440, was estimated to be adequate; assuming the proportion of women of reproductive age group who used FA periconception to be 17% [8] using a precision (d) of 5% and 95% confidence interval. Information obtained with the questionnaire included sociodemographic details of age, occupation, marital status and highest completed level of education; maternal obstetric characteristics such as parity, gravidity, details of antenatal care and time of registration; and use of FA. The women were asked if they ever used FA prior to conception, within the first trimester of pregnancy or at any other time during pregnancy. The time of commencement of FA intake, the doses, the reason for using the FA and information concerning who prescribed the FA use were equally ascertained. They were also asked about their knowledge of dietary fortification with FA. The socioeconomic class of the respondents was obtained using their occupation, based on the Economic and Social Research Council (ESRC) guidelines and categorised into: Class I - managerial and professional, Class II - intermediate and Class III - working class [9].

The responses were computed and data analysed using SPSS version 19 (SPSS Inc, Chicago, IL, USA). Univariate statistical analysis was performed on descriptive variables and results presented as proportions, percentages, medians or means and standard deviations. Responses obtained from continuous variables were categorised for the purpose of bivariate analysis using means (for maternal age), primigravida vs. others (for number of pregnancies to date) and three months cut-off for time of booking for antenatal care. Tests of association between independent variables of preconceptional FA intake/awareness of FA intake for the prevention of birth defects and sociodemographic/obstetric variables were conducted using chi-square statistics. In view of the small number of women who commenced FA during preconception and in order to fit into a predictive model, tests of association were conducted between early exposure to FA (defined in this study as commencement of FA before the end of the first trimester of pregnancy) and sociodemographic/maternal obstetric variables. Variables that were significant at a p-value of 0.2 were then entered sequentially into a multinomial logistic regression model with the reference category being late commencement of FA (i.e. commencing FA at a time outside the first trimester of pregnancy). The p-value for statistical significance was set at 0.05.

## Results

### Sociodemographic characteristics

A total of 602 mothers participated in the study with 340 (56.5%) recruited from the general hospital and 262 (43.5%) from the tertiary hospital. The mean age of the respondents was 29.3 ± 5.0 years (range of 17 to 42 years). Most of the women (587, 97.5%) were married. The majority (398, 66.1%) were in the working class; the rest were either in the managerial and professional socioeconomic class (86, 14.3%) or in the intermediate class (118, 19.6%). A total of 139 (23.0%) respondents had a university degree, 158 (26.2%) had post secondary/high-school education, 302 (50.2%) had between one and twelve years of formal education while 3 (0.5%) had no formal education.

### Maternal obstetric characteristics

Each of the respondents had between one and six pregnancies; 198 (32.9%) were primigravida. The median number of children for each respondent was 2 (range from 1 to 6). The month of registration of the pregnancy of the index baby for antenatal care (ANC) ranged from the first to the eight month (median of five months); only 155 (25.7%) booked within the first three months of pregnancy.

### FA intake

The majority of respondents (559, 92.9%) took FA at some point or the other during pregnancy. The FA was mostly prescribed by a doctor or a nurse (535, 95.7%). Only 15 (2.5%) respondents took FA during preconception. By the end of the first trimester, 163 (29.2%) women had commenced FA intake, i.e. periconceptional, whereas 396 (70.8%) commenced FA at a later date. There were 43 respondents (7.1%) who did not take the supplement at all. Of the 559 respondents who took FA during pregnancy; 17 (3.0%) knew that it could prevent birth defects in addition to its role as a haematinic, 231 (41.3%) knew that it was taken to boost haematocrit or help in maternal well-being during pregnancy while 311 (55.6%) had no idea why it was prescribed to them. Four respondents (0.7%) knew that foods could be fortified with FA and 451 (74.9%), after being provided information about the possibility of such, would recommend strong advocacy for a campaign to have foods fortified with FA in the country, Nigeria.

### Periconceptional FA intake vs. sociodemographic and maternal obstetric characteristics

The proportion of women in the managerial and professional socioeconomic class who took FA periconception was significantly higher than those of women in the intermediate or working classes (55.8%, 33.6% and 21.4% respectively, p < 0.001). Also, a significantly higher proportion of women who had university education took periconceptional FA compared to those with lower levels of education (53.6% vs. 21.1%, p < 0.001). The proportion of primigravid mothers who took folic acid periconception was higher than that of women with more than one previous pregnancy (41.0% vs. 23.4%, p < 0.001). Furthermore, the proportion of respondents who booked for ANC in the first trimester and took FA periconception was higher than that of respondents who booked outside the first trimester of pregnancy but took FA periconception (88.2.2% vs. 6.9%, p < 0.001). There was no association between periconceptional intake of FA and the age or marital status of respondents (Table 1).

**Table 1:** Association between periconceptional folic acid intake and sociodemographic/maternal obstetric characteristics of the participants

Periconceptional folic acid					
Variable	Taken No (%)	Not taken No (%)	Total No (%)	X^2^	p value
**Age (years)**					
< 30	72 (27.0)	195 (73.0)	267 (100.0)	1.190	0.275
≥ 30	91 (31.2)	201 (68.8)	292 (100.0)		
Total	163 (29.2)	396 (70.8)	559 (100.0)		
**Marital status**					
Single	5 (33.3)	10 (66.7)	15 (100.0)	0.130	0.718
Married	158 (29.0)	386 (71.0)	544 (100.0)		
Total	163 (29.2)	396 (70.8)	559 (100.0)		
**Educational status**					
University education	74 (53.6)	64 (46.4)	138 (100.0)	53.089	<0.001*
Others	89 (21.1)	332 (78.9)	421 (100.0)		
Total	163 (29.2)	396 (70.8)	559 (100.0)		
**Socioeconomic class**					
Managerial/Professional	48 (55.8)	38 (44.2)	86 (100.0)	41.194	<0.001*
Intermediate class	38 (33.6)	75 (66.4)	113 (100.0)		
Working class	77 (21.4)	283 (78.6)	360 (100.0)		
Total	163 (29.2)	396 (70.8)	559 (100.0)		
**Gravidity**					
Primigravida	75 (41.0)	108 (59.0)	183 (100.0)	18.415	<0.001*
Multigravida	88 (23.4)	288 (76.6)	376 (100.0)		
Total	163 (29.2)	396 (70.8)	559 (100.0)		
**Commenced ANC** a **in**					
First trimester	135 (88.2)	18 (11.8)	153 (100.0)	355.9	<0.001*
Outside first trimester	28 (6.9)	378 (93.1)	406 (100.0)		
Total	163 (29.2)	396 (70.8)	559 (100.0)		

* Statistically significant

a ANC – antenatal care, FET – Fisher's Exact Test used when Chi square was inappropriate

### Knowledge of the role of FA vs. sociodemographic and maternal obstetric characteristics

Of the respondents in the managerial and professional socioeconomic class, 11.6% knew that FA could prevent birth defects compared to 0.9% of those in the intermediate class and 1.7% in the working class (p < 0.001). A total of 15 women out of 138 (10.9%) with a university degree who used FA in pregnancy were aware of its preventive role in the aetiopathogenesis of birth defects compared to 2 of the 421 (0.5%) women with less education (p < 0.001). Again, a higher proportion of respondents who booked for ANC in the first trimester knew that FA could be taken to prevent birth defects than those who registered their pregnancies at a later date (5.9% vs. 2.0%, p = 0.016). There was no association between awareness of the preventive role of FA and; respondent's age, marital status and number of pregnancies (Table 2).

**Table 2:** Relationship between awareness of folic acid for the prevention of birth defects and sociodemographic/maternal obstetric characteristics of the participants

Awareness of preventive role of folic acid					
Variable	Aware No (%)	Not aware No (%)	Total No (%)	X^2^	p value
**Age (years)**					
< 30	9 (2.4)	258 (97.6)	267 (100.0)	0.188	0.664
≥ 30	8 (2.6)	284 (97.4)	292 (100.0)		
Total	17 (3.0)	542 (97.0)	559 (100.0)		
**Marital status**					
Single	1 (6.7)	14 (93.3)	15 (100.0)	0.687	0.407
Married	16 (2.9)	528 (97.1)	544 (100.0)		
Total	17 (3.0)	542 (97.0)	559 (100.0)		
**Educational status**					
University education	15 (10.9)	123 (89.1)	138 (100.0)	38.083	<0.001*
Others	2 (0.5)	419 (99.5)	421 (100.0)		
Total	17 (3.0)	542 (97.0)	559 (100.0)		
**Socioeconomic class**					
Managerial/Professional	10 (11.6)	76 (88.4)	86 (100.0)	25.593	<0.001*
Intermediate class	1 (0.9)	112 (99.1)	113 (100.0)		
Working class	6 (1.7)	354 (98.3)	360 (100.0)		
Total	17 (3.0)	542 (97.0)	559 (100.0)		
**Gravidity**					
Primigravida	8 (4.4)	175 (95.6)	183 (100.0)	1.633	0.201
Multigravida	9 (2.4)	367 (97.6)	376 (100.0)		
Total	17 (3.0)	542 (97.0)	559 (100.0)		
**Commenced ANC** ^a^ **in**					
First trimester	9 (5.9)	144 (94.1)	153 (100.0)	5.767	0.016*
Outside first trimester	8 (2.0)	398 (98.0)	406 (100.0)		
Total	17 (3.0)	542 (97.0)	559 (100.0)		

* Statistically significant

a ANC – antenatal care

### Predictors of late exposure to FA in pregnancy

The results of the multinomial logistic regression analysis for the determinants of late exposure to FA in pregnancy among this study population showed that: (i) respondents in the working socioeconomic class were more likely to be exposed to FA later than recommended in pregnancy compared to those in higher social classes (OR = 4.29, CI: 1.59, 11.31, p = 0.004), (ii) women without university degrees were more likely than those with university education to commence FA late in pregnancy (OR = 4.58, CI: 3.06, 6.87, p < 0.001), and (iii) those who booked later in pregnancy were also more likely to commence later than others (OR = 104.27, CI: 53.09, 204.76, p < 0.001). Maternal age and the number of previous pregnancies, however, did not significantly predict time of commencement of FA in this study population (Table 3).

**Table 3:** Logistic regression analysis of relationship between late exposure to folic acid and sociodemographic/maternal obstetric variables

Variable	Categories	OR	95% CI	p value
**Age group**	< 30 years	1.739	0.239 – 3.650	0.144
	≥ 30 years			
**Socioeconomic class**	Working class	4.238	1.588 – 11.313	0.004*
	Intermediate class	1.159	0.481 – 2.789	0.742
	Professionals/managers			
**Educational status**	No university education	4.584	3.058 – 6.871	< 0.001*
	University education			
**Booking for ANC**	Outside 1^st^ trimester	104.267	53.094 – 204.763	< 0.001*
	Within 1^st^ trimester			
**Number of pregnancies**	Multigravida	1.995	0.951 – 4.185	0.068
	Primigravida		

* Statistically significant

## Discussion

This study was conducted on a sample of women from different socioeconomic strata representative of a typical developing country. There was a fairly high proportion in the working class, only 23% had a university degree and 75% booked for antenatal care in the second or third trimester of pregnancy [7]. Among this cohort, there is a low prevalence of use of FA during preconception. Only 2.5% of the respondents used the supplement as recommended - daily ingestion of FA prior to pregnancy and well into the third month of conception [3, 4]. Also, only about the same proportion was aware of the role of periconceptional FA in the prevention of birth defects. This prevailing level of knowledge and practice is extremely low when compared to 37% usage during preconception in the Netherlands [10], 30% - 43% in Australia [11], 31% in Israel [12], and 33% - 44% in the United States [13, 14]. The level of awareness, and, usage are, however, similar to what had been reported from other low resource settings such as 5% awareness of the timing of supplementation in Nepalese women [15]. The relatively low rate of utilization of antenatal care services in African and other developing countries [8] may be partly responsible to this abysmal uptake of a beneficial behaviour. This is further confirmed by the low proportion of women (25%) that had registered for antenatal care services by the end of the first trimester. Furthermore, there is no nationally funded campaign or programme on FA supplementation preconception in the country unlike what obtains for iodine and vitamin A fortification by relevant groups in most developing countries [15].

Periconceptional FA intake is more likely to be seen in women in higher socioeconomic classes, those with university degrees and who booked early for antenatal care. Knowledge of the role of FA in prevention of birth defects when taken periconception was equally better in these groups of women. Similar pattern of behaviour has been reported among Dutch women, in a study in which those with higher educational attainments regardless of ethnicity were more likely to use FA periconception [10]. Education and social class would seem to play important roles in both usage and awareness of benefits of periconceptional intake of FA because pregnancy tends more likely to be planned by women in these categories. Unplanned pregnancies, which are more often recorded in women of lower socioeconomic classes, those with lower levels of education and those who booked late for pregnancy care have been associated with poorer use of FA periconception [10, 16, 17]. It is also probable that women in higher socioeconomic classes or with better education have greater access to information. They are thus more empowered, and more likely to practice safer health behaviours. Planning pregnancy is important since women usually become aware of their pregnancy about three weeks after becoming pregnant, when FA supplementation may be too late to prevent neural tube defects [15, 18]. That only 44% of the women interviewed knew that FA has benefits as a haematinic may be an evidence that antenatal care counselling is still inadequate in the country. Coupled with the absence of organised screening services for congenital malformations [19], this portends problems in the attempt to reduce the rising incidence of NTDs in poorer nations. Mandatory fortification of grains and other food sources with FA has been suggested as a way of improving the primary preventive efforts. This has resulted in a significant decline in the incidence of NTDs in countries that have implemented such programmes [20]. Although only four women knew that such a programme can be implemented, 75% of the others after a short counselling were highly supportive of pursuing a strong advocacy campaign in this respect.

A major limitation of the present study is that it relied on the recollection ability of the mothers as to the details of their use of FA before or during pregnancy. However, in order to minimise the effects of such recall bias, we excluded women whose children were delivered over 11 months before the interview. Another main limitation of this work is the fact that it is not a community or population survey. It is only a hospital-based study whose outcome may not be easily extrapolated to the general population. However, the study was conducted in a highly cosmopolitan Nigerian urban state capital, and the two health facilities chosen for this study were the main obstetric care centres in the city with the various social strata represented.

## Conclusion

In conclusion, periconceptional FA intake in Nigeria is poor amongst mothers generally and particularly significant among those in the working class, those less educated, multigravid or late ANC attendees. The same groups of women are less likely to know about the role of FA in the prevention of birth defects.
